# SCARA5 plays a critical role in the commitment of mesenchymal stem cells to adipogenesis

**DOI:** 10.1038/s41598-017-12512-2

**Published:** 2017-11-01

**Authors:** Hyemin Lee, Yoo Jeong Lee, Hyeonjin Choi, Jo Woon Seok, Bo Kyung Yoon, Daeun Kim, Ji Yoon Han, Yoseob Lee, Hyo Jung Kim, Jae-woo Kim

**Affiliations:** 1 Department of Biochemistry and Molecular Biology, Integrated Genomic Research Center for Metabolic Regulation, Institute of Genetic Science, Yonsei University College of Medicine, Seoul, 120-752 Korea; 2 Department of Integrated OMICS for Biomedical Sciences, Graduate School, Yonsei University, Seoul, 120-749 Korea; 3 Division of Metabolic Disease, Center for Biomedical Sciences, National Institutes of Health, Cheongju-si, Chungbuk 28159 Korea; 4 Brain Korea 21 PLUS Project for Medical Science, Yonsei University, Seoul, 120-752 Korea

## Abstract

Mesenchymal stem cells have the capacity to give rise to multiple cell types, such as adipocytes, osteoblasts, chondrocytes, and myocytes. However, the molecular events responsible for the lineage specification and differentiation of mesenchymal stem cells remain unclear. Using gene expression profile studies, we determined that Scavenger receptor class A, member 5 (SCARA5) is a novel mediator of adipocyte commitment. SCARA5 was expressed at a higher level in committed A33 preadipocyte cells compared to C3H10T1/2 pluripotent stem cells. Gain- and loss-of-function studies likewise revealed that SCARA5 acts as a mediator of adipocyte commitment and differentiation in both A33 and C3H10T1/2 cells. RNAi-mediated knockdown of SCARA5 in A33 cells markedly inhibited the adipogenic potential, whereas overexpression of SCARA5 enhanced adipocyte differentiation in C3H10T1/2 cells. We also demonstrated that the focal adhesion kinase (FAK) and ERK signaling pathways is associated with the SCARA5-mediated response, thereby modulating adipocyte lineage commitment and adipocyte differentiation. Additionally, glucocorticoids induced the expression of SCARA5 in differentiating adipocytes through glucocorticoids response elements (GRE) in the SCARA5 promoter. Taken together, our study demonstrates that SCARA5 is a positive regulator in adipocyte lineage commitment and early adipogenesis in mesenchymal stem cells.

## Introduction

Obesity is a growing health concern and a major risk factor for chronic diseases, including type 2 diabetes, hypertension, and atherosclerosis^1,2^. Obesity leads to an increase in adipose tissue mass that not only enlarges adipocytes (hypertrophy) but also increases cell numbers (hyperplasia)^3^. Adipocyte differentiation is controlled by the coordinated actions of many transcription factors, including members of the CCAAT/ enhancer-binding protein (C/EBP) family and peroxisome proliferator activated receptor-γ (PPARγ)^4–6^. Generally, the molecular mechanisms that regulate the differentiation of adipocytes have been elucidated; however, the factors involved in the commitment process remain unclear.

Populations of pluripotent stem cells residing in the stromal vascular fraction (SVF) of adipose tissue and mesenchymal stem cells (MSCs) have the capacity to undergo commitment to several lineages including adipocytes, myocytes, osteocytes, and chondrocytes^7–9^. Further, the development of MSCs into terminally differentiated adipocytes can be divided into four stages: lineage commitment, preadipocyte proliferation, growth arrest, and terminal differentiation. Thus, a multipotent stem cell line could be used to further our understanding of the process of adipocyte commitment. C3H10T1/2 MSCs were originally isolated from C3H mouse embryos^10^; thus, this cell line is suitable for studying the adipocyte commitment process. Bone morphogenetic protein (BMP) 4 and BMP2 reportedly play a role in the commitment of pluripotent stem cells to the adipocyte lineage^8,11–13^. Thus, exposure of dividing C3H10T1/2 stem cells to BMP4 or BMP2 gives rise to preadipocyte-like cells. When treated with differentiation inducers at the growth arrest stage, these cells enter the adipose development pathway, express adipocyte markers, and acquire the adipocyte phenotype. Similarly, the A33 cell line, which was cloned from C3H10T1/2 cells treated with 5-azacytidine, was established to study adipocyte commitment^11^ and was shown to differentiate into adipocytes in the absence of exogenous BMP2/4 treatment.

In the present study, we used the C3H10T1/2 and A33 cell lines and microarray analysis to determine that Scavenger receptor class A, member 5 (SCARA5) is a potential critical regulator in adipogenic commitment. A previous report indicated that SCARA5 acts as a tumor suppressor by binding focal adhesion kinase (FAK) and that the interaction inhibits the activation of the FAK-Src-Cas pathway, which is linked to the development and progression of hepatocellular carcinoma^14^. Increased SCARA5 expression causes the inactivation of STAT3, a key transcriptional regulator in pro-inflammatory gene expression^15^. SCARA5 expression can also confer the cellular recognition of bacterial pathogens^16^. In addition, SCARA5 reportedly functions as a renal receptor for ferritin during endocytosis and delivers this iron-containing ligand to specific kidney tissues^17^. However, the specific role of SCARA5 in the preadipocyte commitment process, which includes MSC commitment and terminal differentiation into adipocytes, remains undefined.

We found that SCARA5 is highly expressed in white adipose tissue and is a novel mediator of adipocyte commitment. The expression of SCARA5 is enriched in preadipocytes and upregulated during the early stage of MSC adipogenesis. These observations suggest that SCARA5 is required for the commitment process, during which it plays an important role in the differentiation of pluripotent stem cells into adipocytes. To this end, we demonstrated that SCARA5 has an essential role in adipocyte lineage commitment via the inactivation of the FAK-ERK signaling pathway. Furthermore, we characterized a molecular mechanism for the induction of SCARA5 expression by glucocorticoids during adipogenesis. These findings suggest that SCARA5 is a mediator of glucocorticoid effects in adipocyte lineage commitment and adipogenesis.

## Results

### SCARA5 is expressed in the SVF of white adipose tissue

**Figure 1:**
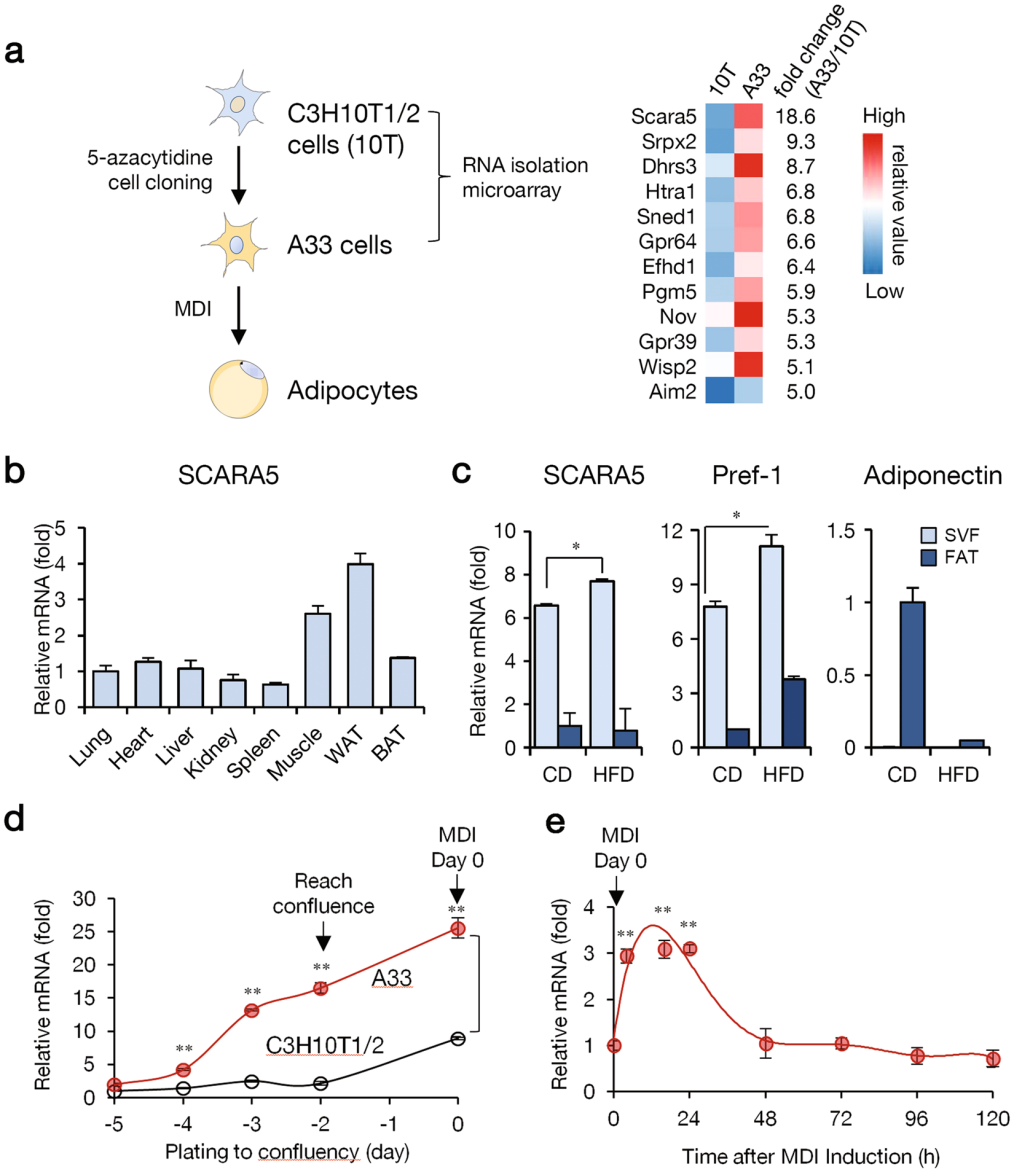
SCARA5 is expressed in the stromal vascular fraction of white adipose tissue. (**a**) The C3H10T1/2 mesenchymal stem cell line and A33 committed stem cell line were compared to identify genes involved in adipocyte commitment. The genes with highly altered expression following commitment (indicated by fold change between A33 and C3H10T1/2 cells) are shown. The total gene expression dataset is available in Supplementary dataset 1. (**b**) Tissue distribution of SCARA5 expression using quantitative real-time PCR analysis (n = 3). (**c**) Real-time PCR of SCARA5 mRNA expression in the stromal vascular fraction (SVF) or fat fraction (FAT) prepared from the epididymal white adipose tissue of mice fed normal chow or a high-fat diet (HFD) (n = 3). (**d**) A33 and C3H10T1/2 cells were grown until post-confluence, total RNA was extracted, and real-time PCR was conducted to detect SCARA5 expression (n = 3). (**e**) A33 cells were differentiated, total RNA was isolated, and real-time PCR was conducted to detect SCARA5 expression at the indicated time points (n = 3). Quantitative data are presented as the mean ± SD. *P < 0.05; **P < 0.01 compared with the control.

A previous report characterized the preadipocyte A33 cell line, which was cloned from C3H10T1/2 cells treated with 5-azacytidine (Fig. 1a)^11^. To identify the molecular mechanisms controlling preadipocyte commitment, we performed preliminary microarray analysis on proliferating A33 and C3H10T1/2 cells and identified *SCARA5*, a novel gene with increased expression in preadipocyte A33 cells compared to C3H10T1/2 cells (Fig. 1a, Supplementary dataset 1). To gain insight into the biological activity of SCARA5, we first examined its expression profile in a wide range of mouse tissues. As expected, SCARA5 was highly expressed in white adipose tissue (Fig. 1b), suggesting the involvement of SCARA5 in adipogenesis. Adipose progenitor cells are closely associated with the adipose SVF and can proliferate and differentiate into mature adipocytes^18^. Thus, we isolated the SVF and the fat fraction from the adipose tissue of mice fed normal chow or a high-fat diet (HFD) to determine the SCARA5 mRNA expression profile. The results revealed that SCARA5 was more abundant in SVF compared with the fat fraction (Fig. 1c), indicating that SCARA5 is expressed at a higher level in preadipocytes than in mature adipocytes. It was reported that the expression of preadipocyte factor 1 (pref-1) is high in preadipocytes but decreases during differentiation and is absent in mature adipocytes; thus, pref-1 is used as a marker for preadipocytes^19,20^. Our data indicated that the expression of SCARA5 increases in SVF in a pattern similar to pref-1 (Fig. 1c).

Taking advantage of the ability to compare uncommitted C3H10T1/2 stem cells and committed A33 cells, we examined the role of SCARA5 in the determination of adipocyte lineage. SCARA5 mRNA was highly expressed in the committed A33 preadipocytes until the cells reach confluence. Unlike A33 cells, SCARA5 expression was lower in uncommitted C3H10T1/2 cells, and the expression levels were not significant at confluence (Fig. 1d). We next analyzed the expression of SCARA5 during adipocyte differentiation after treatment with hormonal inducers. A33 cells were differentiated into adipocytes using a standard adipocyte differentiation protocol. The expression of SCARA5 was upregulated during the early stage of adipogenesis in A33 cells but decreased thereafter (Fig. 1e). Consistent with the adipogenic phenotype, the expression of C/EBPβ, C/EBPα, PPARγ2, aP2/422, and FAS was upregulated during A33 cell differentiation (Supplementary Fig. S1). Taken together, these results indicated that SCARA5 is not expressed in uncommitted MSCs but is abundant in committed preadipocytes and that its expression is regulated during the early stage of adipogenesis.

### Knockdown of SCARA5 severely impairs adipocyte differentiation and its overexpression induces differentiation

**Figure 2:**
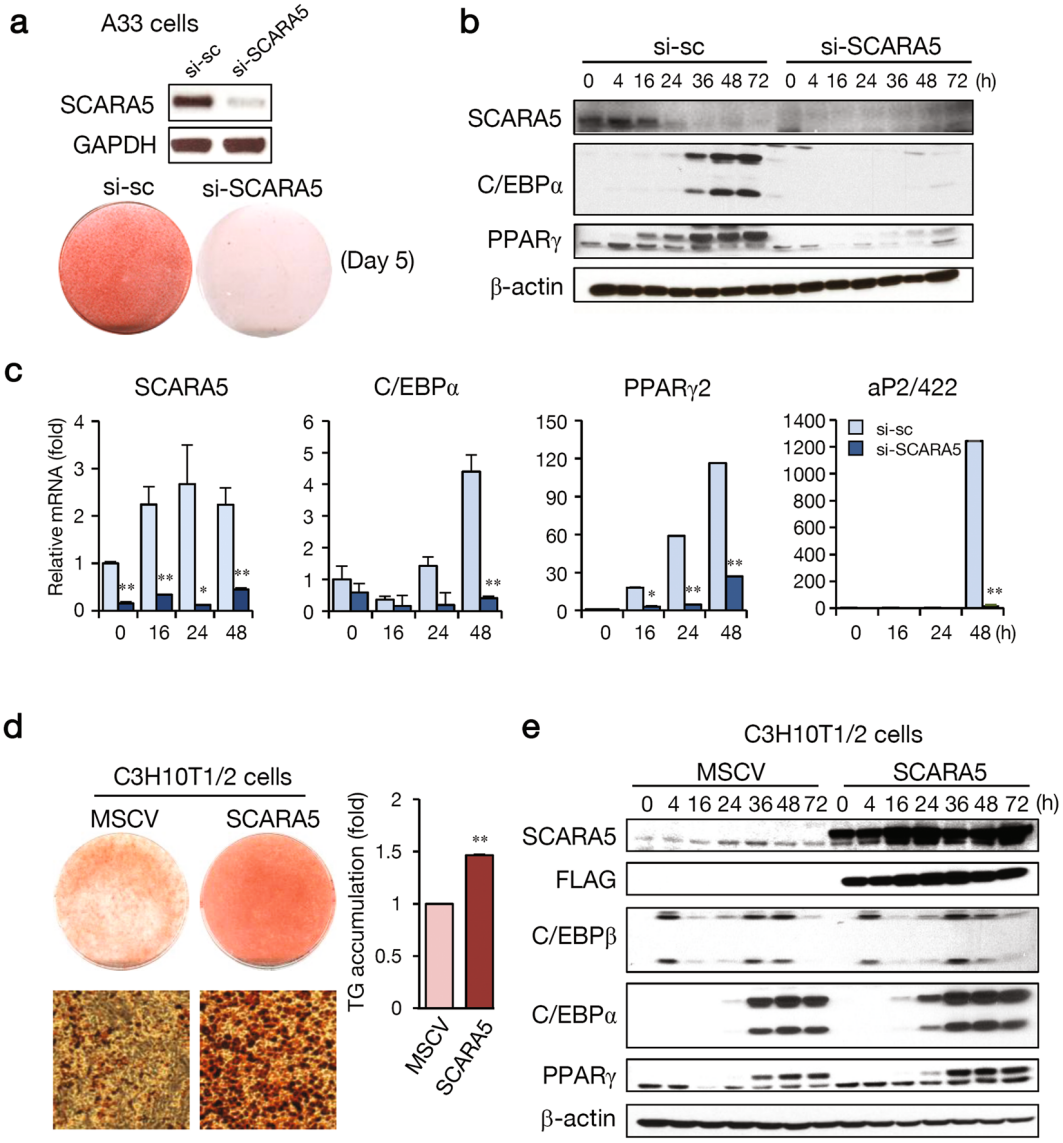
Knockdown of SCARA5 inhibits the commitment to adipocyte lineage and adipocyte differentiation. (**a**) The effects of SCARA5 silencing on commitment to the adipocyte lineage and subsequent adipocyte differentiation were assessed on day 5 by Oil red-O staining. The efficiency of the siRNA knockdown was assessed by RT-PCR on day 2 after transfection. (**b**) Proteins collected at the indicated times after induction were used to assay SCARA5, C/EBPα, and PPARγ2 levels with β-actin as the loading control. (**c**) SCARA5, C/EBPα, PPARγ2, and aP2/422 mRNA levels were measured in the scramble (sc) or si-SCARA5 cells using real-time PCR. (**d**) C3H10T1/2 cells were infected with MSCV or MSCV-SCARA5. After reaching post-confluence, cells were induced to differentiate using an adipocyte differentiation protocol and Oil red-O staining and triglyceride content was measured using spectrometric analysis on day 5 following induction. (**e**) Cells were harvested at the indicated times, and western blotting analysis was performed for SCARA5, FLAG, C/EBPβ, C/EBPα, and PPARγ using β-actin as the loading control. Quantitative data are presented as the mean ± SD (n = 3). *P < 0.05; **P < 0.01 compared with the control.

To evaluate whether SCARA5 affects adipogenesis, we knocked down the expression of SCARA5 in A33 cells using siRNA. Knockdown inhibited adipocyte differentiation, as shown by Oil red-O staining (Fig. 2a). The expression of adipogenic genes (C/EBPα and PPARγ) was significantly reduced in the SCARA5-knockdown cells (Fig. 2b). In addition, mRNAs for C/EBPα, PPARγ2, and aP2/422 were reduced in SCARA5-knockdown cells (Fig. 2c). Because the abundance of SCARA5 was specific to proliferating A33 cells, we hypothesized that the introduction of SCARA5 into C3H10T1/2 cells might bias the cells toward a preadipocyte lineage. We constructed a SCARA5-overexpresssing murine stem cell virus (MSCV) promoter-SCARA5 plasmid by cloning SCARA5 into the MSCV vector and transfected the plasmid into C3H10T1/2 cells. Exogenously expressed SCARA5 in C3H10T1/2 cells significantly increased the differentiation of adipocytes and the triglyceride contents (Fig. 2d). Increased adipogenesis was associated with a significant increase in adipogenic marker expression in SCARA5-overexpressing cells (Fig. 2e). These results suggest that the overexpression of SCARA5 promotes the adipogenic potential of C3H10T1/2 cells. Conversely, C3H10T1/2 cells treated with BMP4, which leads to adipogenic commitment^11^, were subjected to SCARA5 knockdown. As shown in Supplementary Fig. S2, adipocyte differentiation was significantly impaired in SCARA5-knockdown C3H10T1/2 cells as indicated by Oil red-O staining and real-time PCR analysis of adipocyte-specific markers.

In another widely used preadipocyte cell model, 3T3-L1 cells, the similar expression pattern of SCARA5 gene was also observed during undifferentiated and differentiated states like A33 cells (Supplementary Fig. S3). Knockdown inhibited adipocyte differentiation, as shown by Oil red-O staining (Supplementary Fig. S3). The SCARA5 knockdown results in decreased cell counts at day 2 and the expression of adipogenic genes (C/EBPα, C/EBPβ and PPARγ) were significantly reduced in the SCARA5-knockdown (Supplementary Fig. S3). Thus, the combined results from the gain- and loss-of-function studies demonstrate that SCARA5 acts as an adipogenesis promoting factor.

### The function of SCARA5 in adipocyte lineage commitment involves FAK-ERK pathway

**Figure 3:**
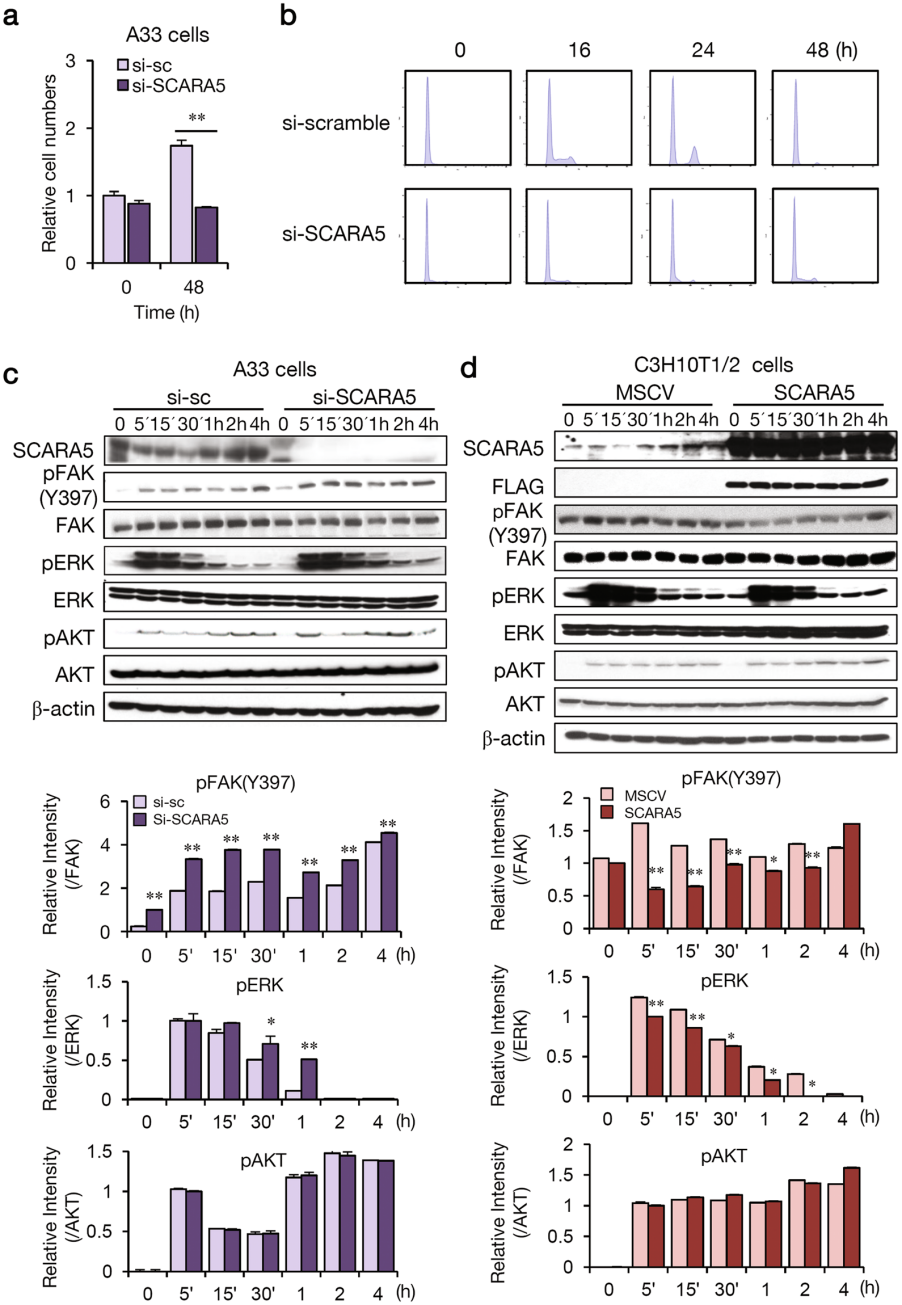
The function of SCARA5 in adipocyte lineage commitment involves FAK-ERK. A33 cells were transfected with si-scramble and si-SCARA5 and differentiated using IBMX, insulin, and dexamethasone (MDI). (**a**) Cell counts were determined at 0 and 48 h after induction. **P < 0.01. Quantitative data are presented as the mean ± SD (n = 3). (**b**) DNA contents were analyzed using flow cytometry at the indicated time points. The suppression of SCARA expression abolished mitotic clonal expansion with a notable decrease in the entry of the cells to the G1-S phase. (**c**) SCARA5, pFAK (Tyr397), FAK, pERK, ERK, pAKT, and AKT were detected by western blotting at the indicated time points. The relative intensity of the western blots was determined in three independent experiments. (**d**) C3H10T1/2 cells were infected with MSCV or MSCV-SCARA5. After reaching post-confluence, cells were induced to differentiate using an adipocyte differentiation protocol. The expression of SCARA5, FLAG, pFAK (Tyr397), FAK, pERK, ERK, pAKT, and AKT were detected using western blotting at the indicated time points with β-actin as a loading control. The relative intensity of the western blots was determined in three independent experiments.

To explore the molecular mechanisms underlying the role of SCARA5 in adipocyte lineage commitment, the activity of potential signaling pathways was evaluated. MSC commitment to the adipose cell lineage yields preadipocytes that, when appropriately induced, undergo mitotic clonal expansion and differentiate into adipocytes^21^. We wondered if cells in the si-SCARA5 group underwent mitotic clonal expansion following inhibition of differentiation. Knockdown of SCARA5 inhibited mitotic clonal expansion (Fig. 3a) based on cell number, which is an indicator of the extent of clonal expansion. In addition, SCARA5-knockdown cells did not enter the G1- to- S stages (Fig. 3b), indicating that SCARA5 is required for cellular signaling during the period between adipogenic determination and early adipogenesis. Previously, it was reported that SCARA5 downregulated the FAK-Src-Cas signaling pathway^14,22^. FAK-mediated adhesive signaling during matrix production and osteogenesis involves ERK activation^23,24^. Additionally, FAK promotes the osteogenic differentiation of MSCs^23,25^. Thus, we investigated if SCARA5 influences the FAK-ERK signaling pathway during adipocyte lineage commitment. To examine the relationship between the function of SCARA5 and FAK in adipogenesis, we performed a knockdown of SCARA5 in A33 cells. As expected, SCARA5 knockdown significantly increased FAK phosphorylation levels for 4 h (Fig. 3c), and there was a clearly detectable increase in ERK phosphorylation. However, pAKT levels were unaffected. Conversely, the overexpression of SCARA5 in the cells decreased FAK phosphorylation and ERK levels (Fig. 3d). ERK has been shown to be necessary for initiating differentiation of preadipocytes, and this signal transduction pathway must be inhibited for adipocyte maturation to proceed^26^. Our results showed that the activation of ERK was markedly prolonged in SCARA5-knockdown cells compared with control cells. These changes in ERK may be associated with the function of SCARA5 during adipogenesis. In addition, we found that phosphorylation of ERK was increased during differentiation and was sustained for 16 h in A33 cells (Supplementary Fig. S4). Conversely, the overexpression of SCARA5 in C3H10T1/2 cells decreases phosphorylated form of FAK protein and ERK levels (Supplementary Fig. S4). Because FAK reportedly promotes the osteogenic differentiation of MSCs^23,25^, SCARA5-mediated suppression of FAK might be required for the differentiation of stem cells into an adipogenic lineage but not an osteogenic one. To investigate this hypothesis, SCARA5- overexpressed cells were subjected to osteogenic differentiation by the treatment with BMP2 in combination with osteogenic medium. As shown in Supplementary Fig. S5, osteogenesis was significantly reduced with reduced expression of osteogenic genes (Runx2, ALP and osteocalcin) in the SCARA5-overexpression cells, suggesting that SCARA5 might function as an anti-osteogenic factor. Taken together, these data indicate that SCARA5 promotes commitment to the adipocyte lineage by regulating the FAK-ERK pathway in A33 cells.

### Dexamethasone induces SCARA5 gene expression during adipocyte lineage commitment

**Figure 4:**
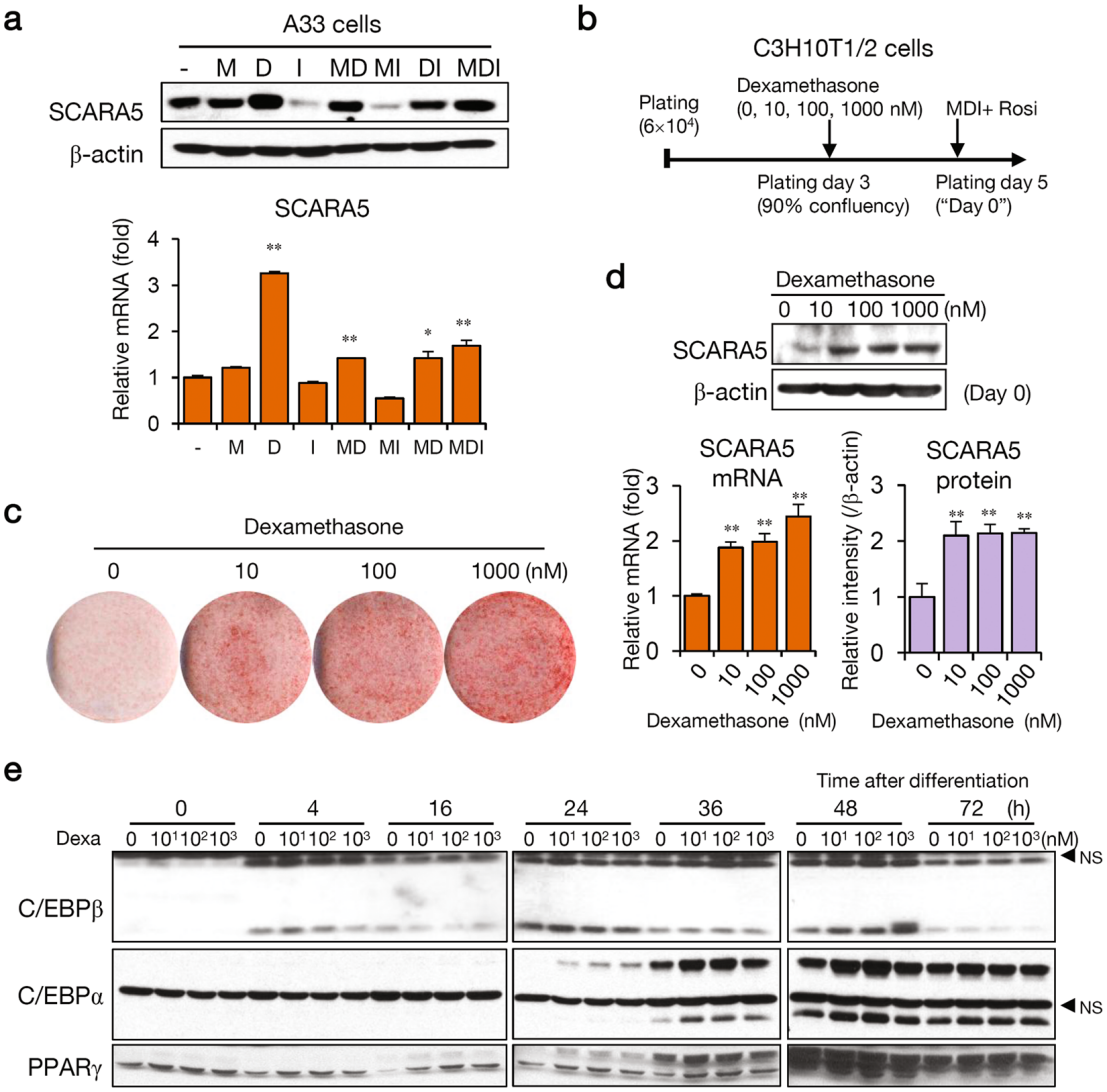
Dexamethasone induces SCARA5 during adipocyte lineage commitment. (**a**) A33 cells were treated for 24 h with individual components of the differentiation cocktail (-, without MDI; M, IBMX; D, dexamethasone; I, insulin). Western blotting (upper) and real-time PCR (lower) were performed to confirm the expression of SCARA5. Quantitative data are presented as the mean ± SD (n = 3). **P < 0.01 compared with the control. (**b**) SCARA5 was induced using the modified protocol in proliferating C3H10T1/2 cells treated with dexamethasone. Dexamethasone treatment was performed during the proliferative stage, and MDI and rosiglitazone were administered 2-days post-confluence, designated as Day 0. (**c**) The cells were stained with Oil red-O after 7 days of differentiation. (**d**) SCARA5 protein and mRNA were detected by western blotting (upper) and real-time PCR analysis (lower left). The relative intensity of the western blots was determined in two independent experiments (lower right). Quantitative data are presented as the mean ± SD (n = 3). **P < 0.01 compared with the control. (**e**) Cells pretreated with dexamethasone were differentiated using differentiation medium. The cells were harvested at the indicated times, and western blotting of C/EBPβ, C/EBPα, and PPARγ was performed (^*^NS, non-specific bands).

SCARA5 expression increased during the differentiation of A33 preadipocytes to mature adipocytes when exposed to a cocktail containing dexamethasone, insulin, and 3-isobutyl-1-methylxanthine (IBMX) (Fig. 1e). This result indicates that one or more of these components induces SCARA5 expression. A previous study reported that glucocorticoid signaling leads to the commitment state during adipogenesis *in vitro* ^27^, and another study reported that SCARA5 expression is upregulated by dexamethasone treatment^28^. We explored if the expression of SCARA5 depends on dexamethasone treatment during early-stage adipocyte differentiation. To determine which components of the differentiation cocktail induced SCARA5 expression, 2-day post-confluence A33 cells were treated for 24 h with the individual components or with the complete cocktail. SCARA5 was induced by dexamethasone but was not upregulated by the other components (Fig. 4a), suggesting that dexamethasone is a major factor in the induction of SCARA5. Because SCARA5-expressing cells promoted the commitment to the adipocyte lineage, we hypothesized that uncommitted C3H10T1/2 cells pretreated with dexamethasone during proliferation would commit to the adipocyte lineage. Thus, we examined if dexamethasone induced the commitment of C3H10T1/2 cells in a dose-dependent manner during proliferation. Cells were treated with dexamethasone at concentrations ranging from 10 nM to 1 µM for 2 days (Fig. 4b). Treatment with dexamethasone prior to confluency promoted commitment of the C3H10T1/2 cells to the adipocyte lineage (Fig. 4c). The expression of SCARA5, both for mRNA and for protein, was upregulated by dexamethasone in a dose-dependent manner (Fig. 4d). During C3H10T1/2 cell differentiation, SCARA5 levels increased a pattern similar to those of the adipocyte markers C/EBPα and PPARγ. The level of C/EBPβ was unaffected (Fig. 4e). These results confirmed that induction of SCARA5 by dexamethasone is required for both adipocyte lineage commitment and early adipogenesis.

### Dexamethasone activation of the SCARA5 promoter is mediated by the glucocorticoid response element (GRE) motif

**Figure 5:**
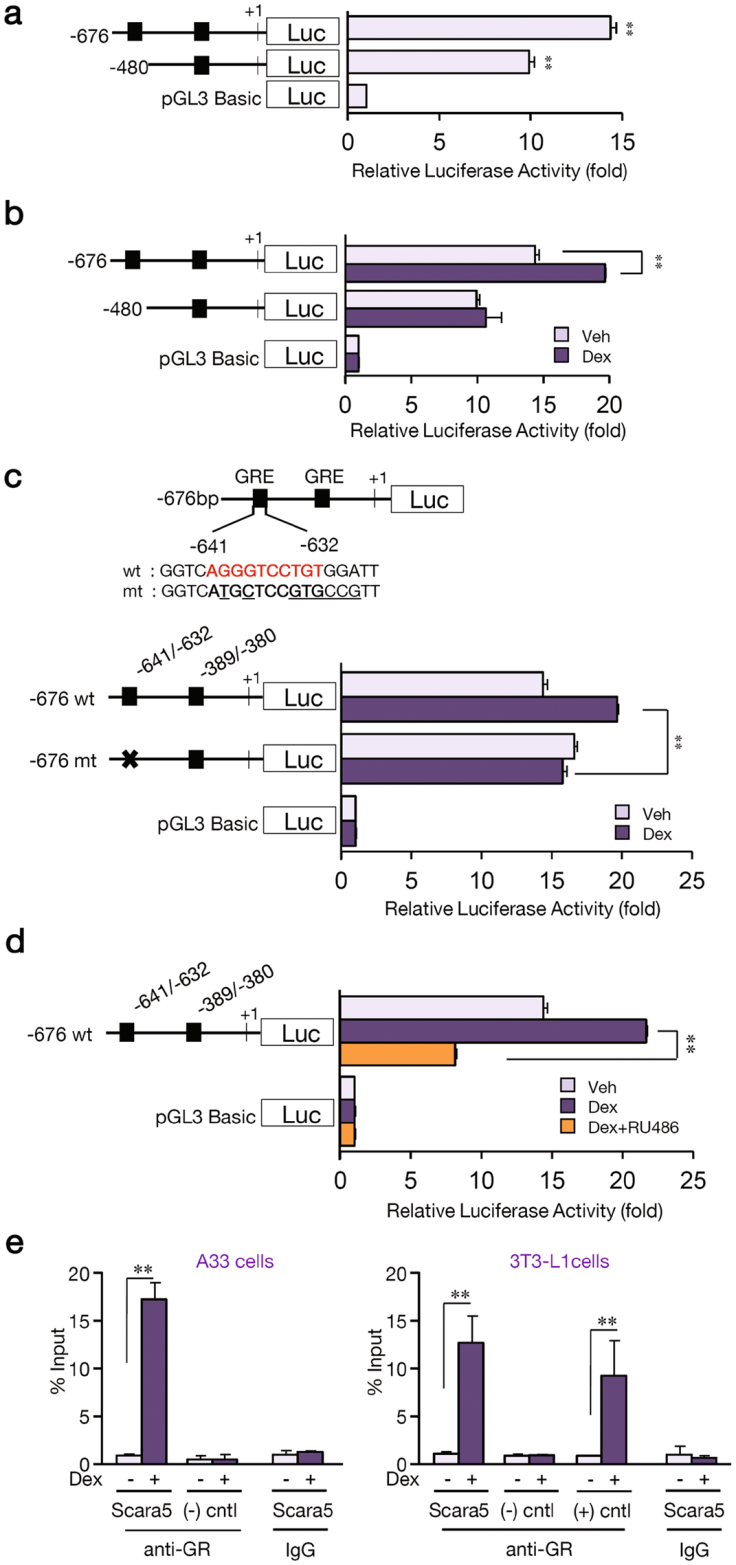
Dexamethasone activation of the SCARA5 promoter is mediated by a glucocorticoid response element (GRE) motif. (**a**) *SCARA5* promoter-luciferase constructs under basal conditions (10% CS). C3H10T1/2 cells were co-transfected with *SCARA5* promoter-luciferase constructs and a Renilla-luciferase control vector. Promoter activity was normalized to Renilla luciferase activity. (**b**) The effect of 2 μM dexamethasone (Dex) on the activity of the *SCARA5*-luciferase constructs in C3H10T1/2 cells. Luciferase activity was measured 24 h after treatment with vehicle or dexamethasone. (**c**) The sequence of the *SCARA5* promoter region containing the GRE (−641 to + 49). The wild-type (wt) putative GRE motif is marked in bold and the mutant (mut) GRE (−641 to −632) sequences are underlined. (**d**) The results of co-treatment with 1 μM RU486 on 2 μM dexamethasone-induced *SCARA5* promoter-luciferase constructs containing wild-type and mutant versions of the GRE. (**e**) ChIP assay was performed using anti-GR antibodies or rabbit IgG. The promoter region (−641 to −632) of SCARA5 with putative GRE was amplified by real-time PCR. The positive control is Dexras1 promoter^44^, and the negative control is >1 kb upstream of SCARA5 promoter. Quantitative data are presented as the mean ± SD (n = 3). *P < 0.05; **P < 0.01 compared with the control.

To verify that increased expression of SCARA5 was induced by dexamethasone, we performed a sequence analysis and discovered putative GREs on the proximal *SCARA5* promoter. A luciferase assay showed increased activity of the *SCARA5* promoter in dexamethasone-treated cells. To evaluate these putative regulatory elements, we cloned a *SCARA5* promoter fragment (−676 to + 49) into a luciferase reporter plasmid. From this parent construct, we generated a series of deletion mutants and luciferase activity was measured 48 h after transfection. As shown in Fig. 5a, the promoter activity was 10~13-fold higher than that of the basic pGL3 vector. To identify functional GREs, cells transfected with the reporter constructs were treated with dexamethasone or vehicle and relative expression levels were determined. The sequences between −676 and + 49 conferred dexamethasone responsiveness, whereas a deletion to −480 abolished this response (Fig. 5b). To evaluate the function of this element, mutations were generated within the putative GRE at −641 to −632. As expected, the wild-type sequence promoted the induction of luciferase activity in response to dexamethasone (Fig. 5c). Conversely, the GRE mutations in −641 to −632 abolished dexamethasone responsiveness. To define the increased luciferase activity conferred by the −676 region, transfected cells were co-treated with dexamethasone and the glucocorticoid receptor-antagonist RU486. As expected, RU486 significantly attenuated dexamethasone-mediated induction (Fig. 5d). To determine whether GR binds to the native SCARA5 promoter, we performed ChIP assays using anti-GR antibodies. The binding of GR was enhanced substantially in response to dexamethasone treatment in SCARA5 promoter in A33 and 3T3-L1 cells (Fig. 5e). Taken together, these findings suggest that GREs were required for the activation of SCARA5 expression mediated by dexamethasone.

## Discussion

The increase in adipose tissue mass associated with obesity is due in part to an increase in adipocyte formation from MSCs^29,30^. Thus, identifying the factors that regulate adipogenesis should provide insight into the mechanisms by which pluripotent MSCs undergo commitment to the adipose lineage. In this study, we searched for candidate genes to identify novel factors that regulate commitment to the adipocyte lineage and early adipogenesis. Previous studies reported that BMP4 could regulate adipocyte lineage commitment^8,11,31^, but other factors that regulate adipocyte commitment and related signaling pathways have not yet been elucidated.

In this study, microarray analysis was used to identify genes that were differentially expressed between uncommitted C3H10T1/2 cells and committed A33 cells, with the goal of identifying genes related to adipocyte lineage commitment. We detected increased SCARA5 expression in preadipocytes as well as in the SVF of adipose tissue. Functional studies indicated that SCARA5 played a critical role during adipocyte lineage commitment, as evidenced by the ability of SCARA5 knockdown to block commitment almost completely, whereas exogenously expressed SCARA5 in C3H10T1/2 cells significantly increased adipocyte differentiation. Based on these results, we concluded that SCARA5 is sufficient to induce adipogenic differentiation of MSCs and preadipocytes. Unlike A33 preadipocytes that are committed to the adipocyte lineage and can be differentiated into adipocytes, C3H10T1/2 cells are functionally similar to MSCs that have not undergone commitment and can differentiate into various mesenchymal lineages, including osteoblasts, chondrocytes, and adipocytes^32^. Adipose tissues contain mature adipocytes and several non-adipocyte cell populations, including endothelial, blood, and mesenchymal cells^33^. Many studies indicate that multipotent adipose-derived stem cells are present in the SVF of adipose tissue. To this end, we confirmed that SCARA5 expression was significantly increased in SVF compared to fat. Consistent with these findings, the overexpression of SCARA5 was sufficient to induce the adipogenic commitment of uncommitted C3H10T1/2 cells. Conversely, SCARA5 knockdown markedly impaired adipocyte differentiation, which demonstrated that SCARA5 is primarily associated with adipocyte lineage commitment.

**Figure 6:**
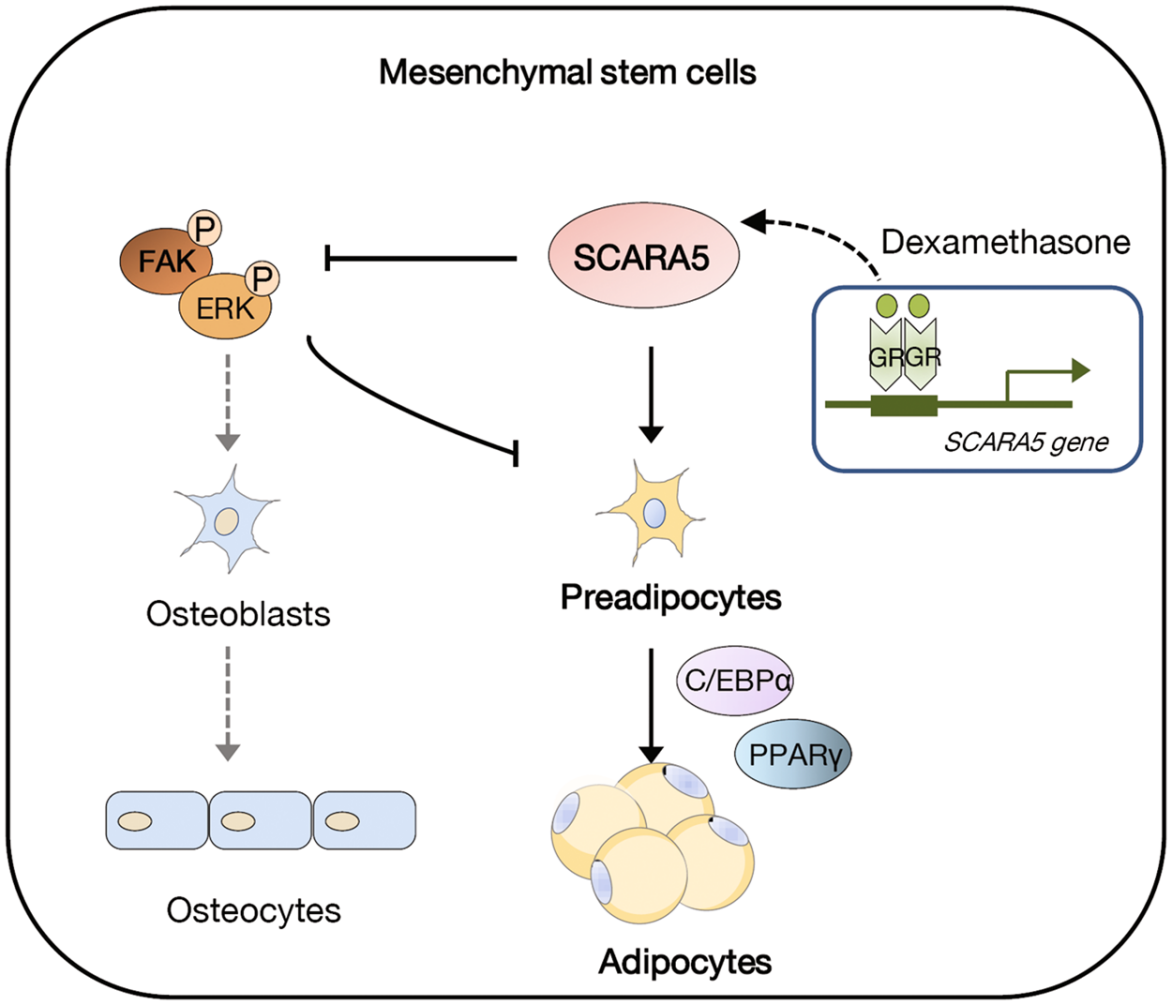
A proposed model of SCARA5 functions in the commitment of mesenchymal stem cells to the adipocyte lineage. In the mesenchymal lineage commitment, dexamethasone induces the expression of SCARA5 through a functional GRE on the promoter. SCARA5 then inactivates FAK signaling, which is reportedly essential for osteogenic differentiation. Through the inactivation of FAK, SCARA5 shifts the fate of mesenchymal stem cells to adipocyte lineage commitment, leading to adipocyte differentiation.

Previous studies have shown that the SCARA5 is associated with a ferritin receptor that mediates the delivery of non-transferrin iron^17^, and that the silencing of SCARA5 might contribute to the activation of FAK signaling in human hepatocellular carcinoma^14^. The Fyn/FAK/mTORC2 signaling pathway decreases adipogenic differentiation of MSCs by enhancing β-catenin signaling and regulates the cytoskeleton by activating RhoA^34^. The activation of FAK is necessary for the osteogenic differentiation of human MSCs^35^ and FAK–Src signaling can activate the mitogen-activated protein kinase (MAPK)/ERK pathway^36^. These reports, combined with our research, indicate that the inhibition of SCARA5 might lead to an increased FAK pathway signaling and attenuate adipogenic differentiation. We found that the knockdown of SCARA5 in A33 cells significantly increased FAK phosphorylation (Fig. 3c), which supports a close relationship between these molecules. When we overexpressed SCARA5 in C3H10T1/2 cells, FAK phosphorylation levels and ERK signaling were significantly decreased (Fig. 3d). A previous investigation indicated that prolonged ERK activation inhibited the early stage of adipogenesis^37^. Another recent report suggested that a disintegrin and metalloproteinase with thrombospondin motifs 1 (*adamts1*) was impaired during adipogenesis when ERK1/2 phosphorylation increased^38^. Our findings indicate that FAK-ERK signaling might be involved in SCARA5-mediated commitment of MSCs, although this correlation may or may not be causative. Nevertheless, this mechanism might contribute to the tight balance by which MSC fate is determined (Fig. 6).

Other studies have revealed that SCARA5 expression was induced by dexamethasone^28^ and during adipogenic development in human MSCs^39^. Similarly, glucocorticoid signaling leads to a commitment state during adipogenesis *in vitro* ^27^. These results led to our hypothesis that an adipocyte induction cocktail containing dexamethasone could regulate the commitment state in the progression from preadipocytes to adipocytes. We determined that dexamethasone induces SCARA5 expression in A33 and C3H10T1/2 cells. In addition, SCARA5 expression was induced when C3H10T1/2 cells were pretreated with dexamethasone during proliferation, which enabled the commitment of C3H10T1/2 cells to preadipocytes. We also identified a functional GRE in an upstream *SCARA5* sequence that confers dexamethasone induction and demonstrated that inactivation of this element abolished the dexamethasone-mediated response. Overall, these findings demonstrate that SCARA5 is a positive regulator of the adipocyte lineage commitment process. The discovery of factors that control SCARA5 expression could further clarify the mechanisms underlying adipogenesis and offer novel insights that could be used to combat obesity and metabolic disease in the modern world.

## Materials and Methods

### Animal experiments

Six-week-old mice were fed normal diet or high-fat diet for 6 weeks and body weight was monitored every week. The composition of the HFD we used was 60 kcal% fat containing 0 g/kg of corn starch, 125 g/kg of maltodextrin 10, 68.8 g/kg of sucrose, and 245 g/kg of lard (Research Diets, New Brunswick, NJ, USA) or normal diet (Dyets, Bethlehem, PA, USA). SVF and mature adipocytes were isolated from epididymal fat pads of 12-week- mice. Briefly, the fat-pads were rapidly excised, finely minced, and incubated at 37 °C for 1 h with 1 mg/ml type I collagenase in Hank’s buffered salt solution containing 1% BSA, 200 nM adenosine, and 50 mg/ml glucose. After sequential filtration and centrifugation, floating adipocytes (fat fraction) and pellet (SVF) were separated. All experimental protocols involving animals, including maintenance and care, were performed in accordance with the National Institutes of Health guidelines and ethics guidelines of Yonsei University, and all animal procedures were approved by the Committee on Animal Investigations of Yonsei University.

### Cell culture and Induction of differentiation

A33 and C3H10T1/2 cells (American Type Culture Collection, Bethesda, MD) were propagated and differentiated as described^40^. A33 cells were cultured and differentiated into adipocytes as described previously^11^. Briefly, A33 and 3T3-L1 cells were maintained in Dulbecco’s modified Eagle’s medium (DMEM) containing 100 U/ml penicillin, 100 μg/ml streptomycin, and 8 μg/ml biotin, supplemented with 10% heat-inactivated calf serum at 37 °C, in an atmosphere of 90% air and 10% CO_2_. To induce differentiation, 2-day post-confluent A33 and 3T3-L1 cells (designated day 0) were incubated in DMEM containing 10% FBS, 0.5 mM 3-isobutyl-1-methylxanthine (IBMX), 1 μM dexamethasone, and 1 μg/ml insulin for 2 days. To commit the cells to adipocyte, C3H10T1/2 cells were plated at a density of 6 × 10^4^ cells/6-cm dish, and 10, 100 and 1,000 nM dexamethasone was added 3 days after plating. For differentiation, 2-day post-confluent C3H10T1/2 cells (designated day 0) were incubated in DMEM containing 10% FBS, 0.5 mM 3-isobutyl-1-methylxanthine, 1 μM dexamethasone, 5 μg/ml insulin, and 1 μM rosiglitazone for 2 days. Cells were then cultured in DMEM containing 10% FBS and 1 μg/ml insulin for another 2 days, after which they were grown in DMEM containing 10% FBS. Oil red-O staining was done at the indicated time points. For induction of osteogenic differentiation, cells were incubated in an osteogenic medium contacting 50 μg/ml ascorbic acid and 5 mM β-glycerophosphate in the presence of 200 ng/ml BMP-2 (Sigma, St. Louis, MO, USA)^41^. The medium was changed to an osteogenic medium. For Alkaline phosphatase staining, cell were washed in PBS, fixed in 3.7% paraformaldehyde for 10 min at room temperature and stained with ALP staining solution containing 0.25% Naphthol ASMX phosphate solution (Sigma) and fast red violet B salt (Sigma).

### Microarray analysis

Microarray analysis was preliminarily performed with C3H10T1/2 and A33 cells using the Affymetrix GeneChip Mouse Gene 1.0 ST Array (DNAlink inc, Korea). RNA samples used had a minimum A260/A280 ratio of > 1.8 and the samples were checked for the integrity. Expression data was analyzed using Affymetrix GCOS software with Robust Multi-array Average (RMA) normalization. The total dataset is available in Supplementary dataset 1.

### Small interfering RNA (siRNA)

The A33 cells were plated into 60-mm-diameter dishes 48 h before transfection. The following double-stranded stealth siRNA oligonucleotides (Invitrogen, Carlsbad, CA, USA) were used: mouse SCARA5, sense 5′-GGGAC CGAAC AGGAC AGCAG AGUGA-3′ and antisense 5′-UCACU CUGCU GUCCU GUUCG GUCCC-3′, a set of three validated siRNA oligonucleotides. Control oligonucleotides with comparable GC content were also from Invitrogen. For knockdown, cells were transfected with control or gene-specific siRNA at 20 nM in OPTI-MEM medium using Lipofectamine RNAiMAX (Invitrogen), according to the manufacturer’s protocol. The next day, the medium was replaced with fresh DMEM containing 10% calf serum, and the cells were incubated for 48 h before the induction of differentiation. Oil red-O staining of SCARA5 knockdown was performed at day 5.

### Retroviral transfection

The cDNA for SCARA5 was generated by PCR using the following primers: 5′-CGCAG ATCTG CCACC ATGGA TTACA AGGAT GACGA-3′ (forward); 5′-GCGAA TTCTC AGGGG ACAGT ACAAG TCA -3′ (reverse). The PCR product was cloned into a MSCV retroviral vector with BglII and EcoRI. 293 T cells cultured in serum-free DMEM were transfected with MSCV or recombinant plasmid at 95% confluence. Fresh medium containing 10% calf serum was given 4~6 h after transfection and the viral medium was collected at 48~72 h. C3H10T1/2 cells were infected with viruses at 20~30% confluence with polybrene (8 μg/ml).

### Gene expression analysis

The level of gene expression was measured by RT-PCR or quantitative real-time PCR using total RNA from A33 cells. Total RNA was isolated from cells or tissues using TRIzol (Invitrogen) according to the manufacturer’s instructions. For RT-PCR, cDNA was synthesized from 5 μg of total RNA using random hexamer primers and SuperScript reverse transcriptase II (Invitrogen). Real time PCR were conducted with SYBR Green PCR Master Mix (Applied Biosystems) using an ABI PRISM 7300 RT PCR system (Applied Biosystems). All data were normalized to 18 s and quantitative measures were obtained using the △△-Ct method (Applied Biosystems). PCR was performed using the following primers: SCARA5, 5′-TGTGG AAGGT TCAGG ATGCG-3′, 5′-GGCTT CGATT GCTTT CCACC-3′; PPARγ2, 5′-TATGG GTGAA ACTCT GGGAG-3′, 5′-GCTGG AGAAA TCAAC TGTGG-3′; C/EBPα, 5′-TGGAC AAGAA CAGCA ACGAG-3′, 5′-TCACT GGTCA ACTCC AGCAC-3′; C/EBPβ, 5′-CAAGC TGAGC GACGA GTACA-3′, 5′-CAGCT GCTCC ACCTT CTTCT-3′; aP2/422, 5′-TCTCC AGTGA AAACT TCGAT-3′, 5′-TTACG CTGAT GATCA TGTTG-3′; FAS, 5′-AAGCC GTTGG GAGTG AAAGT-3′, 5′-CAATC TGGAT GGCAG TGAGG-3′; Pref-1, 5′-GACCC ACCCT GTGAC CCC-3′, 5′-CAGGC AGCTC GTGCA CCCC-3′; Adiponectin, 5′-ATGGC AGAGA TGGCA CTCCT-3′, 5′-CCTTC AGCTC CTGTC ATTCC-3′; GAPDH, 5′-ACCAC AGTCC ATGCC ATCAC-3′, 5′-TCCAC CACCC TGTTG CTGTA-3′. Runx-2, 5′-CCAGG CAGGT GCTTC AGAAC TG-3′, 5′-ACATG CCGAG GGACA TGCCT GA-3′; Alkaline phosphatase (ALP), 5′-TATGG TAACG GGCCT GGCTA C-3′, 5′-TGCTC ATGGA CGCCG TGAAG CA-3′; Osteocalcin (OC), 5′-TGAAC AGACT CCGGC GCTAC-3′, 5′-AGGGC AGCAC AGGTC CTAA-3′. All reactions were performed in triplicate. Relative expression level and S.D values were calculated using the comparative method.

### Western blot analysis and antibodies

For protein analysis, cells were washed with ice-cold PBS and lysed in a buffer containing 1% SDS and 60 mM Tris-Cl, pH 6.8. The lysate was mixed, boiled for 10 min, and centrifuged at 12,000 g for 10 min at 4 °C. Protein concentrations were assessed using the BCA assay kit (Thermo Scientific, Rockford, IL, USA). Protein samples of equal amount were separated by SDS-PAGE and transferred to nitrocellulose membranes. Immunoblot analyses were performed using the following antibodies: polyclonal antibody against C/EBPβ (sc-150), C/EBPα (sc-61), and SCARA5 (sc-98123) (Santa Cruz Biotechnology, Santa Cruz, CA, USA), pFAK (Tyr397, 44-625 G) (Invitrogen, Carlsbad, CA, USA), FAK (3285 S), pERK (9101), ERK (9102), pAKT (Ser473, 4060 S), and AKT (9272 S) antibodies (Cell signaling, Danvers, MA, USA), mouse monoclonal antibody against PPARγ (sc-7273), and β-actin (sc-47778) (Santa Cruz Biotechnology, Santa Cruz, CA, USA), FLAG (F3165) (Sigma, St. Louis, MO, USA).

### Cell count assay

A33 cells were seeded at a density of 1 × 10^4^ cells/well into 24-well plates, incubated for 24 h and synchronized to quiescence by serum starvation for 12 h. At indicated time points, the cells were trypsinized and cell numbers were determined using an automated cell counter, ADAM (NanoEnTek), according to the manufacturer’s instructions.

### FACS analysis

A33 cells were trypsinized and collected by centrifugation, washed with PBS, and then fixed in 90%(v/v) cold methanol. Fixed cells were washed with PBS and stained with 50 μg/ml propidium iodide (PI) and 100 μg/ml RNase A in PBS for 30 min in the dark. Labeled cells were analyzed using a FACS caliber flow cytometry system, and data were analyzed using the ModFit software (BD Biosciences) as described^42^.

### Luciferase assay and Site-directed Mutagenesis

C3H10T1/2 cells were transfected with 0.2 μg pGL3-SCARA5 plasmid, 10 ng pRL-CMV (Promega, Madison, WI, USA), using Lipofectamine (Invitrogen), according to the manufacturer’s instructions. After 24 h, cells were treated with 2 μM dexamethasone of the solvent DMSO in DMEM containing 10% CS. After 24 h, the luciferase activities were measured using the Dual Luciferase Reporter Assay System (Promega), according to the manufacturer’s instructions. Firefly luciferase activities were standardized to Renilla activities. PCR amplification of the wild-type luciferase reporter plasmid was performed using site-directed mutation primers (SCARA5-756mt, 5′-CCCCA GCACT GGAGT GCCAG GTGCG CCCAC CACGC C-3′, 5′-GGCGT GGTGG GCGCA CCTGG CACTC CAGTG CTGGG G-3′; -676mt, 5′-CCTGC TTGAG AGGTC ATGCT CCGTG CCGTT CCACT ATCGC C-3′, 5′-GGCGA TAGTG GAACG GCACG GAGCA TGACC TCTCA AGCAG G-3′).

### Chromatin Immunoprecipitation (ChIP) assay

ChIP assay was performed as described^43^. Briefly, nuclear proteins were cross-linked to genomic DNA with 1% formaldehyde for 10 min at room temperature. Cells were scraped into ice-cold PBS containing protease inhibitors and following centrifugation, pellets were resuspended in lysis buffer (0.1% SDS, 140 mM NaCl, 1 mM EDTA, 1% Triton X-100, 0.1% Na-deoxycholate, 5 μM leupeptin, 2 μM pepstatin, 1 μM aprotinin, 0.5 mM PMSF and 50 mM Hepes, pH 7.9) and incubated on ice for 10 min. Lysates were sonicated and centrifuged after which the resulting supernatants were incubated with rotation in the presence of anti-GR antibody (1 μg). Following the addition of protein A/G-agarose (SantaCruz Biotechnology) reactions were incubated for 4 h at 4 °C and then immune complexes were precipitated by centrifugation. After sequential washes with wash buffer (0.1% SDS, 1% Triton X-100, 1 mM EDTA, 500 mM NaCl, 50 mM Hepes, pH 7.9, and 0.1% Na-deoxycholate, 5 μM leupeptin, 2 μM pepstatin, 1 μM aprotinin, 0.5 mM PMSF) and LiCl wash buffer (250 mM LiCl, 0.5% NP-40, 0.5% Na-deoxycholate, 1 mM EDTA and 20 mM Tris-Cl, pH 8.0), the beads were washed twice with TE buffer. Following centrifugation, the resulting immune complexes were resuspended in 200 μl elution buffer (50 mM Tris-Cl, pH 8.0, 1 mM EDTA, 1% SDS and 50 mM NaHCO_3_) and incubated for 10 min at 65 °C. After another centrifugation step, the supernatant was collected and cross-linking reversed by adding NaCl to a final concentration of 0.3 M. The remaining proteins were digested with proteinase K and genomic DNA fragments recovered by phenol-chloroform extraction, followed by ethanol precipitation and resuspension in sterile H_2_O. Mouse genomic sequences containing the putative glucocorticoid receptor binding sites (GRE) and a region further upstream from the SCARA5 promoter were amplified using the following primers: 5′- TTATG TGGGG GCTGG GGAT -3′ and 5′- ACCTC AACAC AAACA ATTAC CTTG-3′. A positive control for GRE, Dexras1 promoter^44^ was used, and a negative control was > 1 kb upstream of the SCARA5 promoter using the following primers: 5′-AGTGA AGCAT CTGGG CCTCT-3′ and 5′-ACTGC AAATT TGGCC CCCAA-3′.

### Statistical analysis

All results are expressed as mean ± s.d. Statistical comparisons of groups were made using an unpaired Student’s t test or two-way ANOVA.

## Electronic supplementary material

Supplementary Information

Supplementary Dataset 1
