# Supplementary material for: SCARA5 plays a critical role in the commitment of mesenchymal stem cells to adipogenesis
Source: Sci Rep. 2017 Nov 1;7:14833. doi: 10.1038/s41598-017-12512-2 (PMC5665884; doi:10.1038/s41598-017-12512-2)

Supplementary Materials

SCARA5 plays a critical role in the commitment of mesenchymal stem cells to adipogenesis

Hyemin Lee, Yoo Jeong Lee, Hyeonjin Choi, Jo Woon Seok, Bo Kyung Yoon, Daeun Kim, Ji Yoon Han, Yoseob Lee, Hyo Jung Kim, Jae-woo Kim

Contents : Supplementary Figures and Legends (Figures S1-S5), Supplementary Dataset 1 (available in the separate excel file), and raw data for western blots.

Supplementary Figure S1

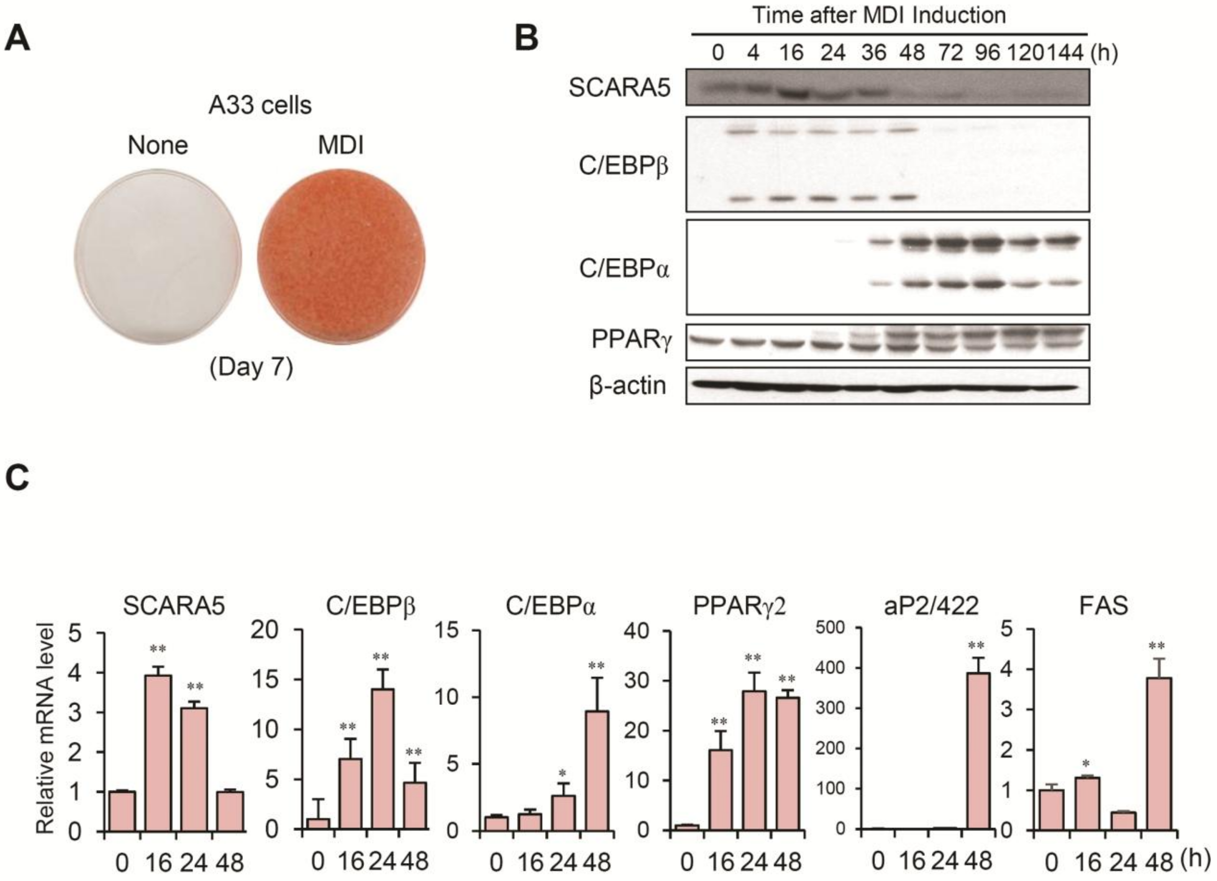

Supplementary Figure S1. The effects of SCARA5 on the differentiation of A33 cells. (**A**) Post-confluence A33 preadipocyte cells were treated with or without differentiation medium. The cells were then stained with Oil red-O on day 7. (**B**) SCARA5 expression during adipogenesis was validated by western blotting. (**C**) SCARA5 expression during adipogenesis was validated using real-time PCR. C/EBPβ, C/EBPα, PPARγ2, aP2/422, and FAS were used as adipocyte differentiation markers. Quantitative data are presented as the mean ± SD (n = 3). *$P < 0.05$; **$P < 0.01$ compared with control.

Supplementary Figure S2

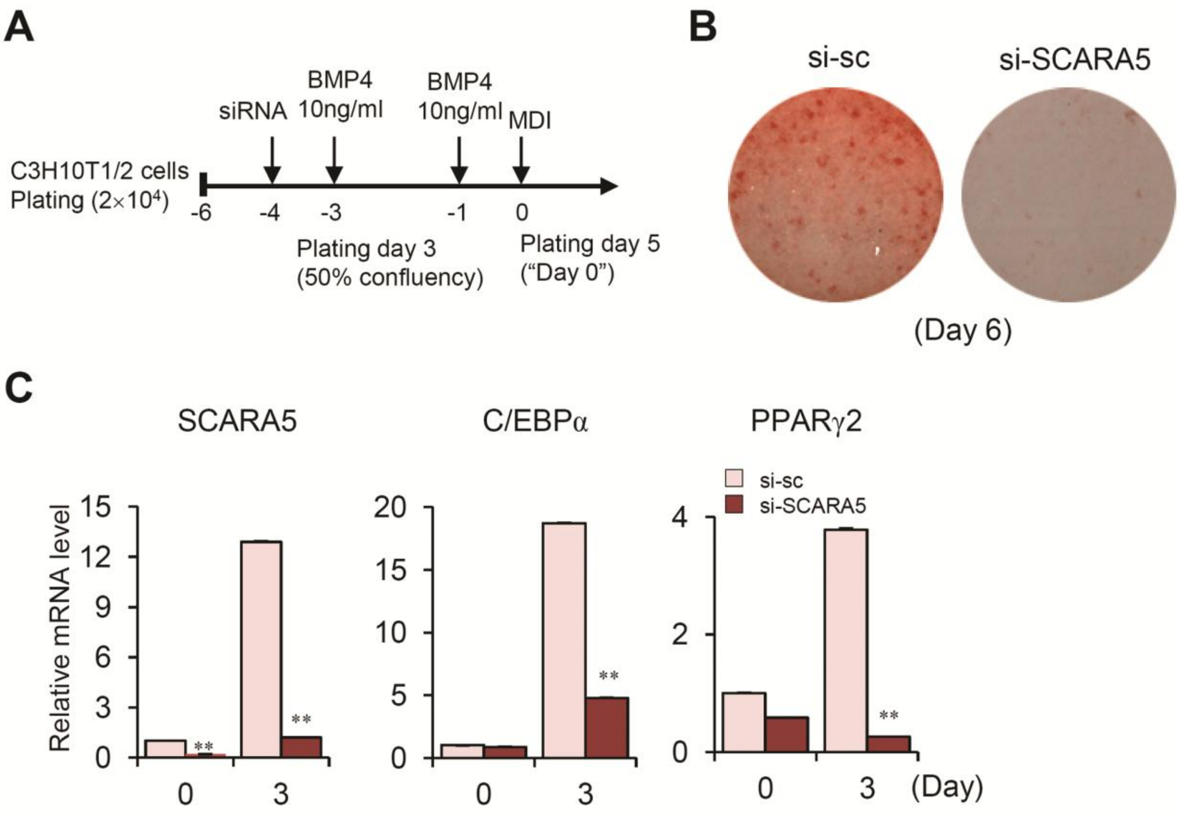

Supplementary Figure S2. Knockdown of SCARA5 inhibits the adipocyte lineage commitment of C3H10T1/2 cells. (**A**) C3H10T1/2 cells were incubated with SCARA5 siRNA or scramble for 24h prior to treatment with BMP4 (10 ng/ml). Cells were grown until postconfluence, and then cell differentiation was induced (MDI). (**B**) The effects of SCARA5 silencing on commitment to the adipocyte lineage and subsequent adipocyte differentiation were assessed on day 6 by Oil red-O staining. (**C**) SCARA5, C/EBPα and PPARγ2 mRNA levels were measured in the scramble (sc) or si-SCARA5 cells using real-time PCR.

Supplementary Figure S3

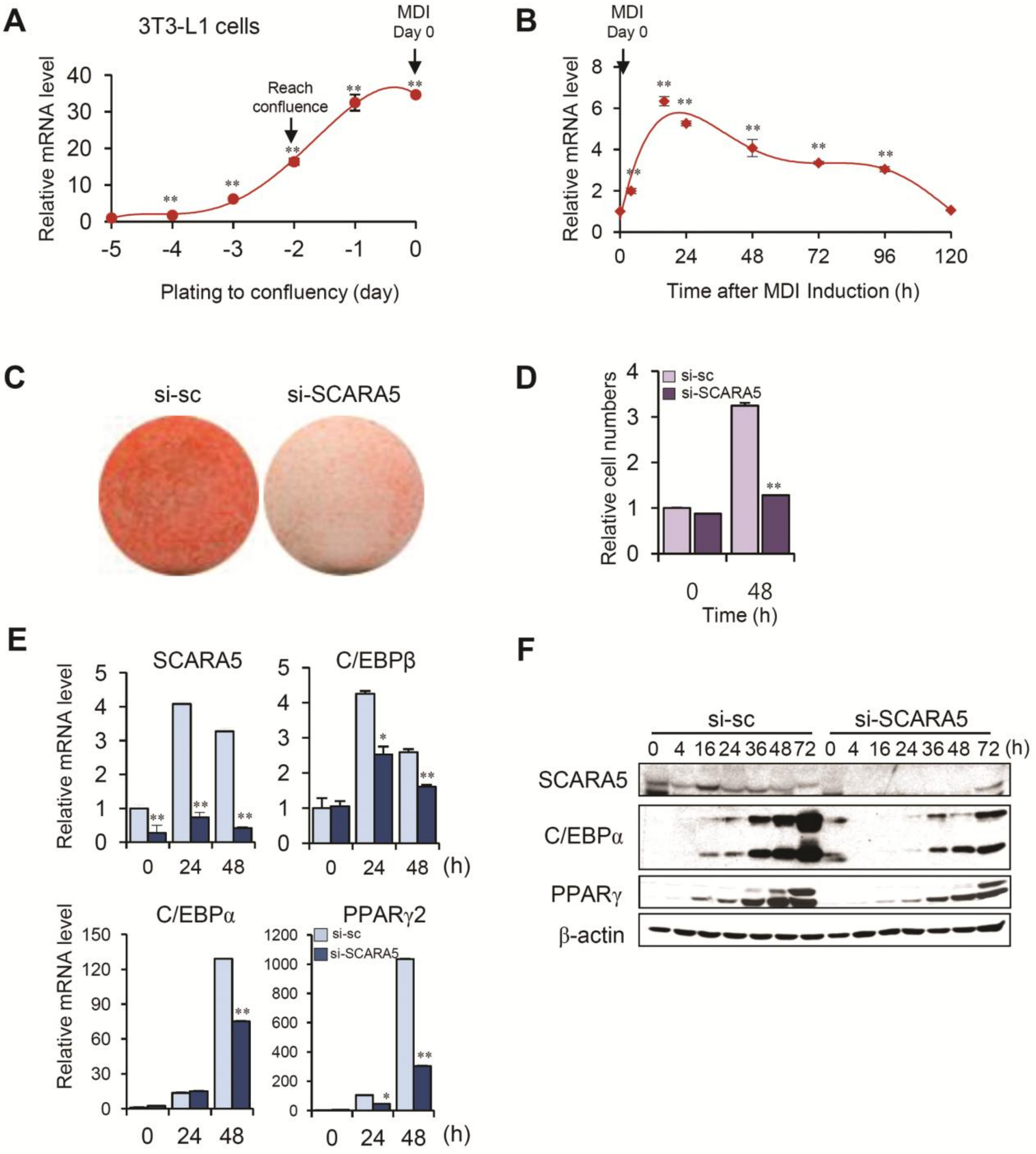

Supplementary Figure S3. Knockdown of SCARA5 inhibits adipocyte differentiation in 3T3-L1 cells. (**A**) 3T3-L1 cells were grown until post-confluence, total RNA was extracted, and real-time PCR was conducted to detect SCARA5 expression. (**B**) 3T3-L1 cells were differentiated, total RNA was isolated, and real-time PCR was conducted to detect SCARA5

expression at the indicated time points. (**C**) The effects of SCARA5 silencing on commitment to the adipocyte lineage and subsequent adipocyte differentiation were assessed on day 5 by Oil red-O staining. (**D**) Cell counts were determined at 0 and 48 h after induction. (**E**) SCARA5, C/EBPα, C/EBPβ and PPARγ2 mRNA levels were measured in the scramble (sc) or si-SCARA5 cells using real-time PCR (**F**) Proteins collected at the indicated times after induction were used to assay SCARA5, C/EBPα, and PPARγ2 levels with β-actin as the loading control. Quantitative data are presented as the mean ± SD (n = 3). **P* < 0.05; ***P* < 0.01 compared with the control.

Supplementary Figure S4

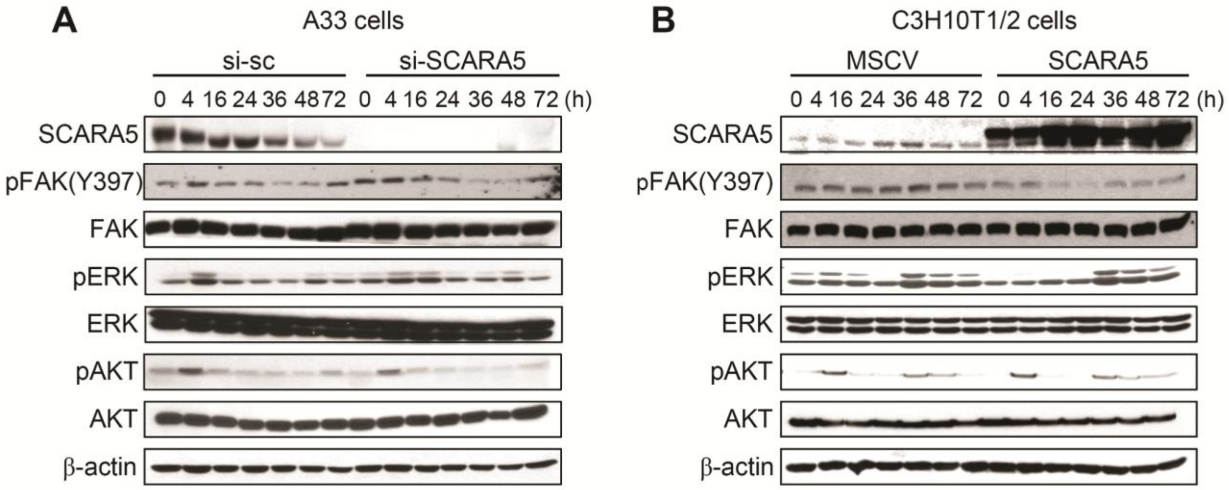

Supplementary Figure S4. The function of SCARA5 in adipocyte lineage commitment is dependent on FAK-ERK. (A) A33 cells were transfected with si-scramble and si-SCARA5 and differentiated using IBMX, insulin, and dexamethasone (MDI). SCARA5, pFAK (Tyr397), FAK, pERK, ERK, pAKT, and AKT were detected at the indicated time points by western blotting with β-actin as a loading control. **(B)** C3H10T1/2 cells were infected with MSCV or MSCV-SCARA5. After reaching post-confluence, cells were induced to differentiate using an adipocyte differentiation protocol. SCARA5, pFAK (Tyr397), FAK, pERK, ERK, pAKT, and AKT were detected at the indicated time points by western blotting with β-actin as a loading control.

Supplementary Figure S5

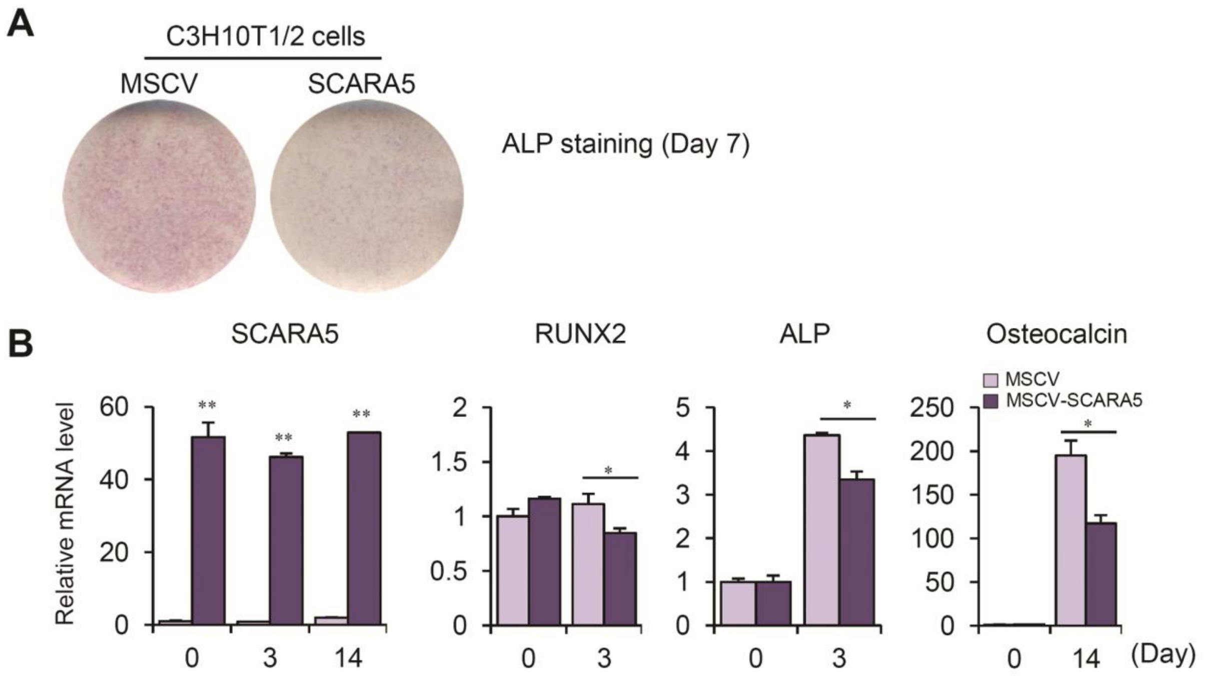

Supplementary Figure S5.The Effects of SCARA5 on osteoblast differentiation in C3H10T1/2 cells. Cells were cultured in an osteogenic medium**.** (**A**) On day 7, cells were fixed and stained with Alkaline phosphatase (ALP) staining. (**B**) Cells were prepared and mRNA of osteoblastic marker genes was analyzed by real-time PCR. Quantitative data are presented as the mean ± SD (n = 3). **P* < 0.05 compared with the control.

Supplementary Dataset 1

Supplementary Dataset 1. Microarray analysis on proliferating A33 and C3H10T1/2 cells (Excel 1.96MB)

Raw data

Fig. 2a

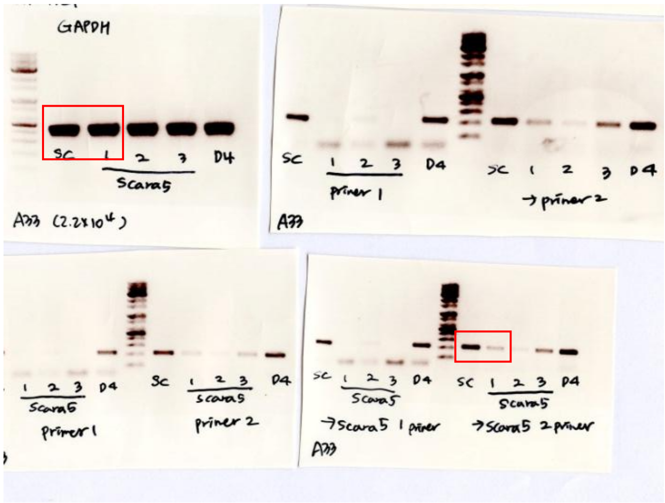

Fig. 2b

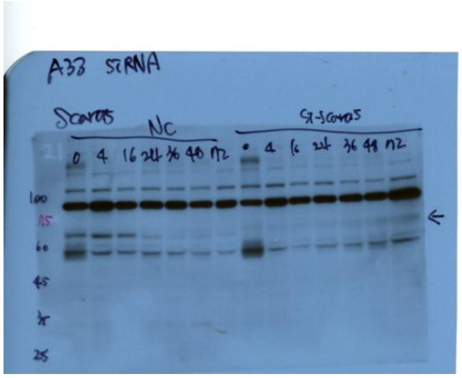
A33 siRNA
NC
100
75
60
45
35
25

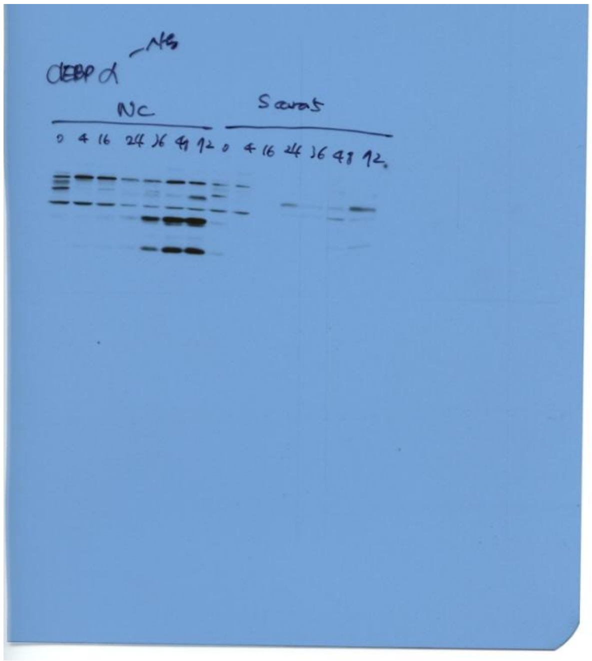
NC
0 4 16 24 36 48 72 0 4 16 24 36 48 72

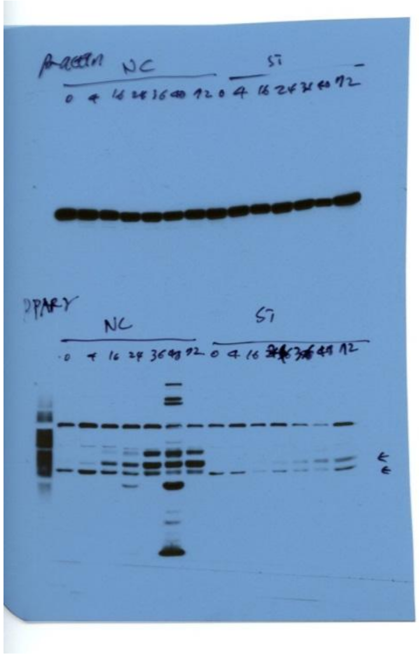
NC
Si
NC
Si

Fig. 2e

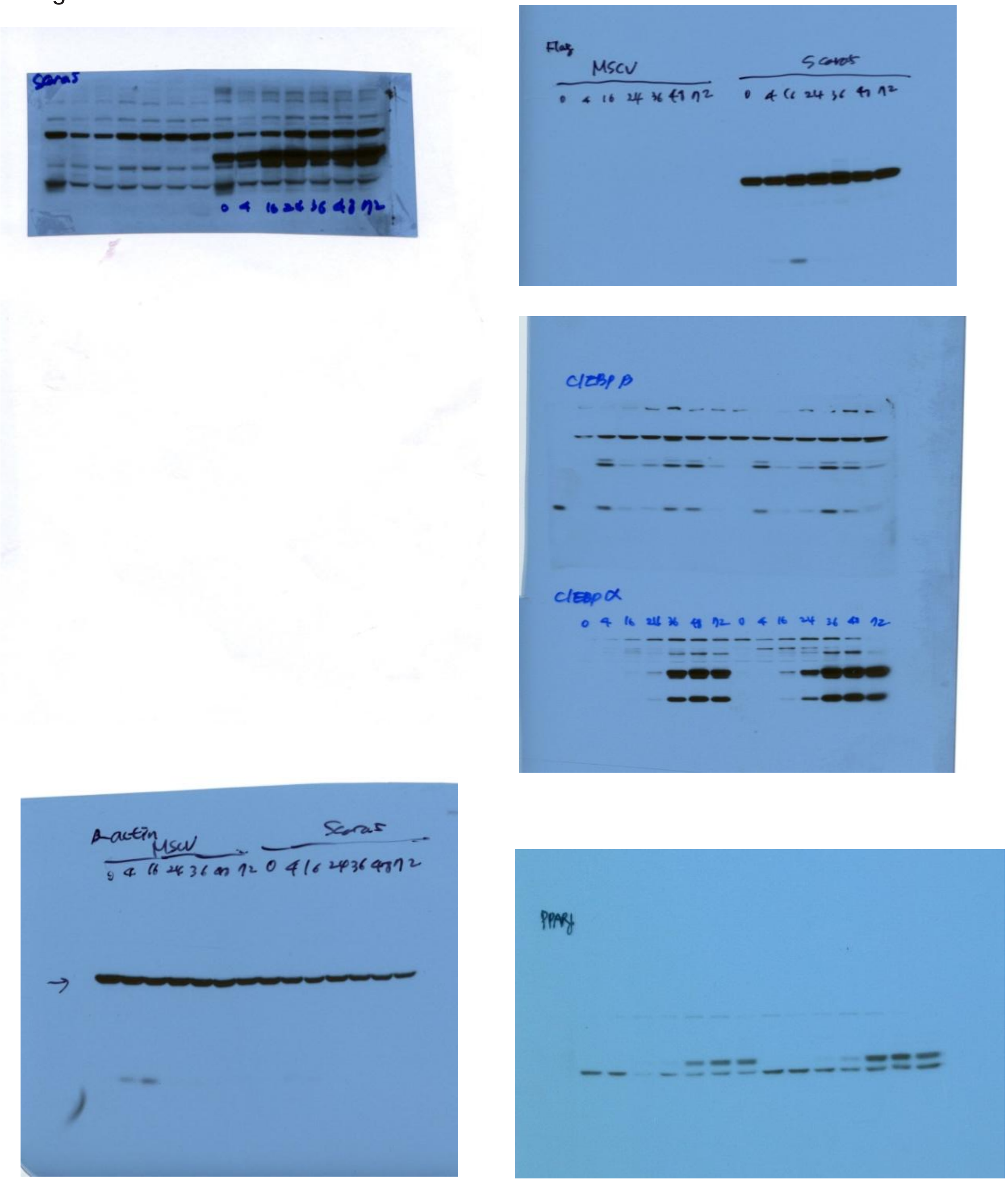

Fig. 3c

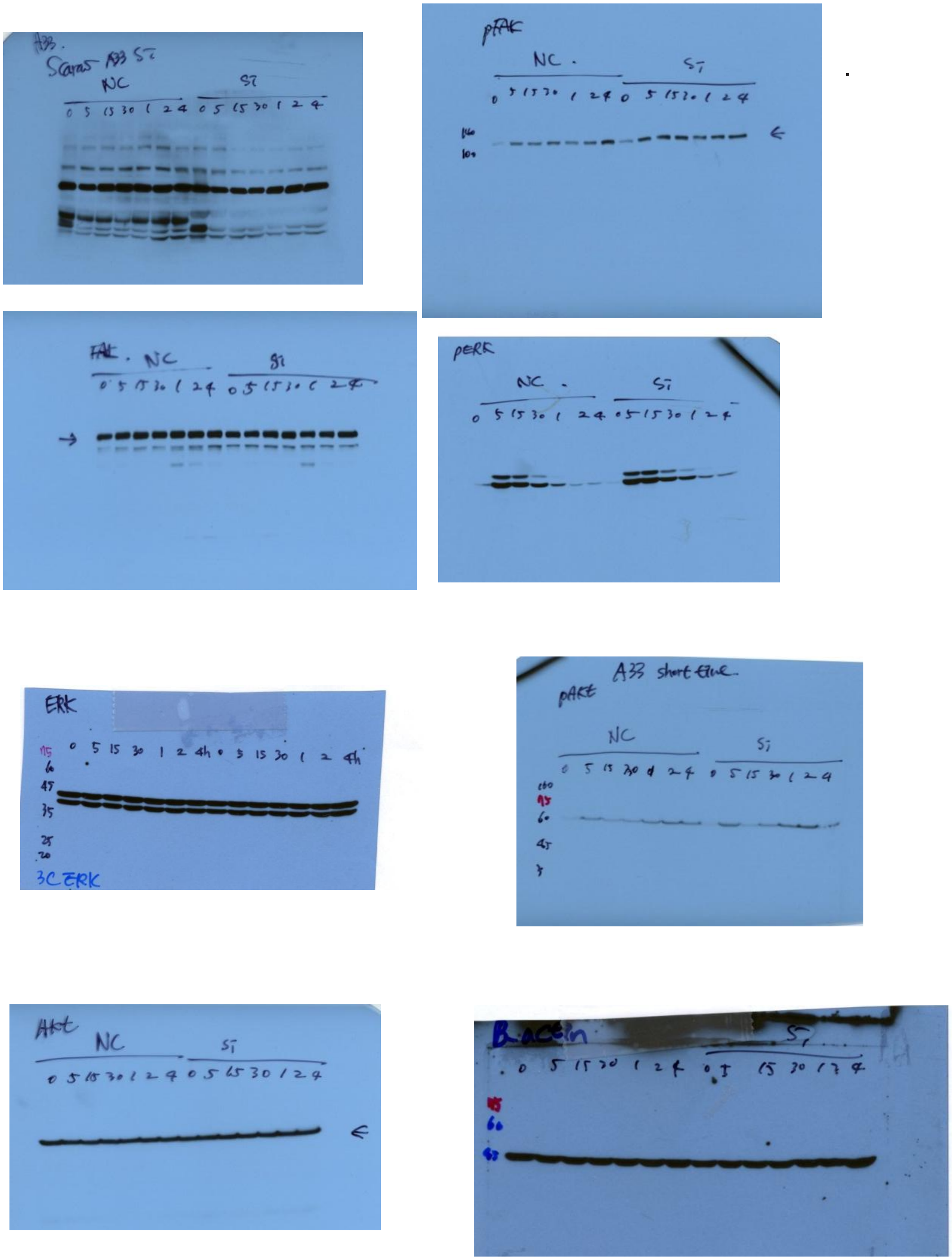

Fig. 3d

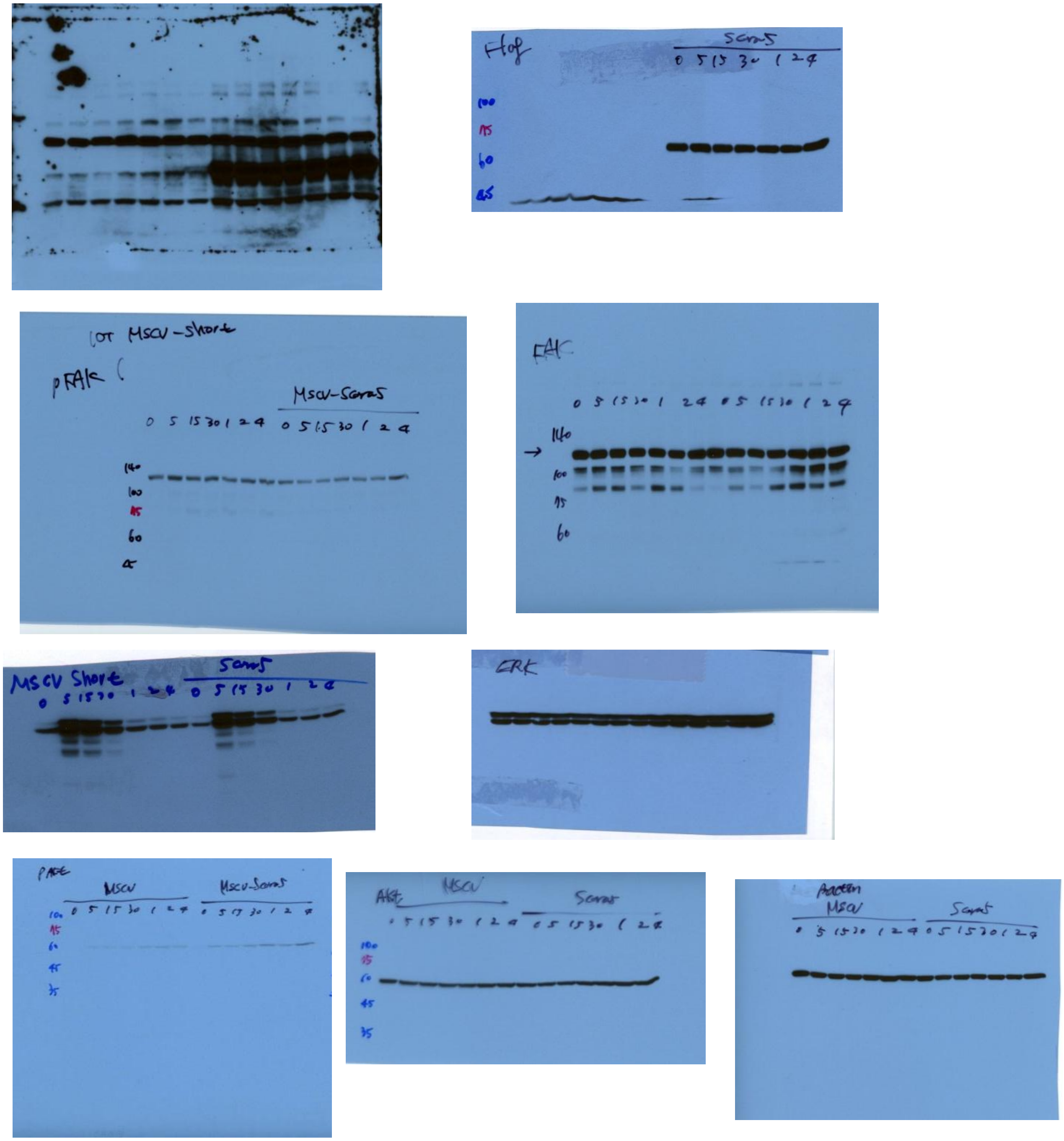

Fig. 4a

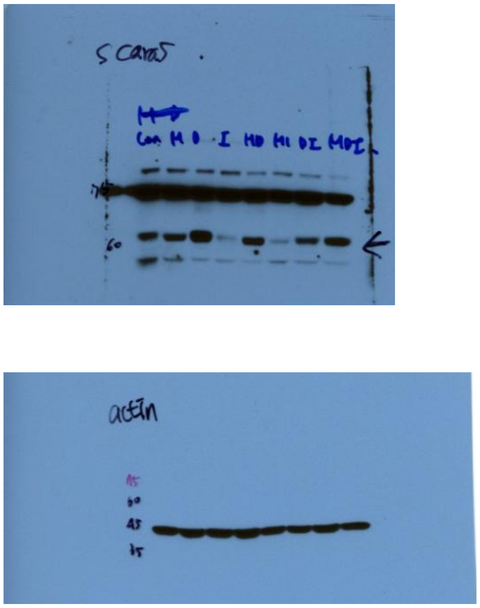

Fig. 4d

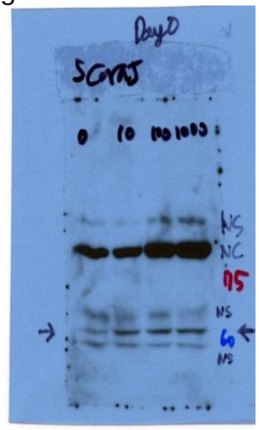
Day0
ScraJ
0
10
100
1000
NS
NC
75
NS
60
NS

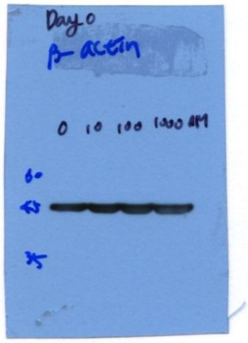
Day 0
β-actin
0
10
100
1000 nM
60
45
35

Fig. 4e

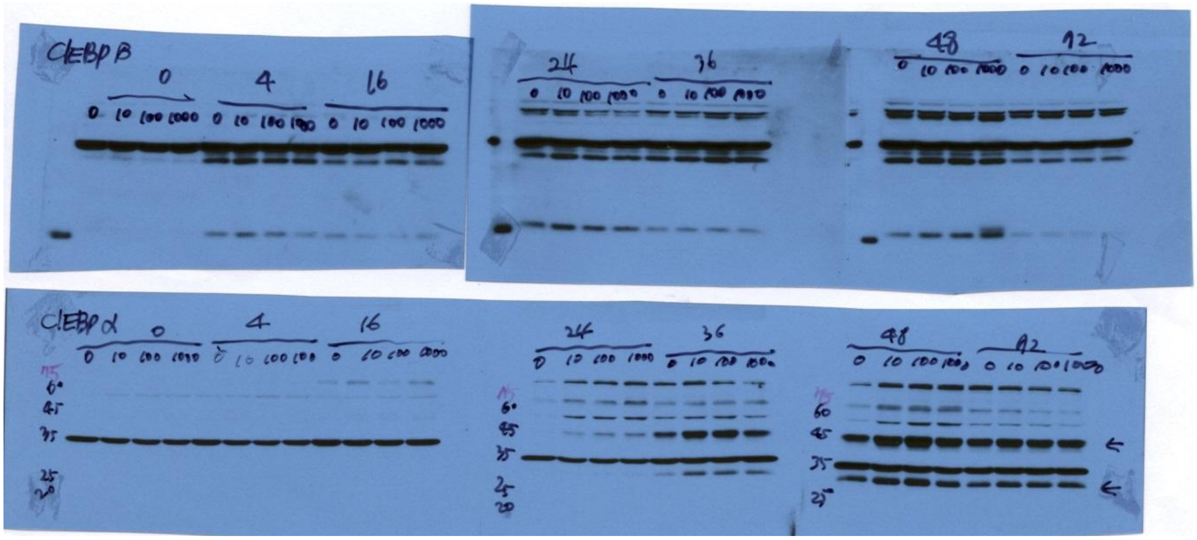

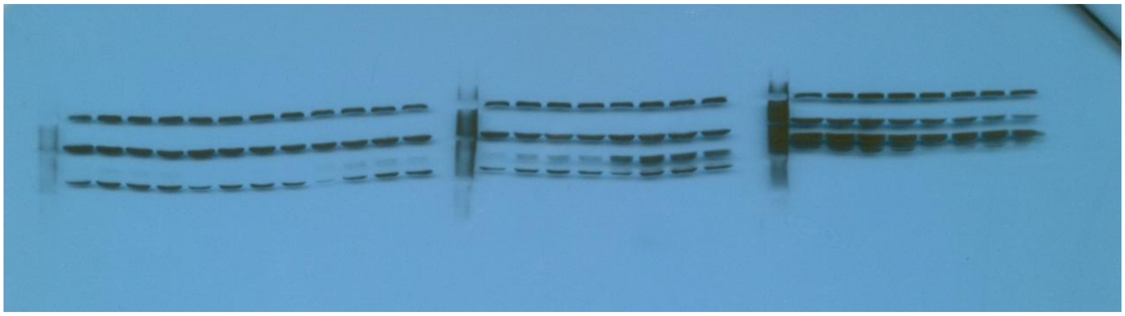

Supplement: Supplementary file 1 — Supplementary Information [file 41598_2017_12512_MOESM1_ESM.pdf]
